# Anticonvulsant, Anticholinesterase and Cytoprotective Effects of the Aqueous Extract of *Lippia sidoides* Cham

**DOI:** 10.3390/plants13213034

**Published:** 2024-10-30

**Authors:** Cicera Janaine Camilo, Débora Odília Duarte Leite, Natália Kelly Gomes de Carvalho, José Walber Gonçalves Castro, Jane Eire Silva Alencar de Menezes, Helcio Silva dos Santos, Joanda Paolla Raimundo Silva, Daniela Ribeiro Alves, Selene Maia de Morais, Fabíola Fernandes Galvão Rodrigues, José Galberto Martins da Costa

**Affiliations:** 1 Federal University of Campina Grande, Campina Grande 58429-900, Paraíba, Brazil; janainecamilo@hotmail.com; 2 Postgraduate Program in Biological Chemistry, Department of Biological Chemistry, Regional University of Cariri, Crato 63105-00, Ceará, Brazil; biodeboraleite@yahoo.com.br (D.O.D.L.); nataliakellygc@gmail.com (N.K.G.d.C.); josewalber@leaosampaio.edu.br (J.W.G.C.); helciodossantos@gmail.com (H.S.d.S.); fabiolafer@gmail.com (F.F.G.R.); 3 Natural Products Research Laboratory, Regional University of Cariri, Crato 63105-00, Ceará, Brazil; 4 Postgraduate Program in Natural Sciences-PPGCN, State University of Ceara, Fortaleza 60714-903, Ceará, Brazil; jane.menezes@uece.br; 5 Multiuser Laboratory of Characterization and Analysis, Federal University of Paraíba, João Pessoa 58051-900, Paraíba, Brazil; joandapaolla.1@gmail.com; 6 Animal Health Research Center—NUPESA, Ceara State University, Av. Dr. Silas Munguba—1700, Campus Itaperi, Fortaleza 60714-903, Ceará, Brazil; alves.danielaribeiro@gmail.com (D.R.A.); selenemaiademorais@gmail.com (S.M.d.M.)

**Keywords:** aqueous extract, epilepsy, Alzheimer’s disease, antihemolytic effect

## Abstract

(1) Background: *Lippia sidoides* Cham is a Brazilian aromatic plant rich in phenolic compounds. In traditional medicine, its leaves are used to treat diseases of the Central Nervous System such as stress and anxiety. This study evaluates the capacity of the aqueous extract of *L. sidoides* as an anticonvulsant, anticholinesterase and antihemolytic agent. (2) Methods: The extract was obtained from the leaves using water as a solvent, then dried in a spray dryer. The anticonvulsant effect was evaluated in zebrafish models using the pentylenetetrazol (PTZ) method. The anticholinesterase effect was determined using the acetylcholinesterase enzyme and physostigmine as a positive control. The antihemolytic action was evaluated by exposing erythrocytes to different concentrations of NaCl in the presence and absence of the extract. (3) Results: The anticonvulsant effect was observed at a concentration of 400 mg/kg, delaying convulsive crises. In the anticholinesterase assay, a dose-dependent action and variation in the effect over time were observed, demonstrating a reversible effect of the extract. For the osmotic fragility test, the extract showed satisfactory results, providing cellular protection across all variations of NaCl concentration. (4) Conclusions: These results demonstrate the promising potential of *L. sidoides* extract for the development of drugs that act in the treatment of diseases that affect the Central Nervous System.

## 1. Introduction

Studies reported in the literature demonstrate that the interaction of plant extracts and isolated compounds with disorders associated with the Central Nervous System (CNS) is common [1,2]. In this context, species of the genus *Lippia* are cited for the treatment of various diseases including those affecting the CNS, associated with the extensive investigation of the chemical composition of essential oils [3]. Despite efforts to chemically characterize and investigate the largest number of biological activities on species of the genus Lippia, there are still limited data related to crude extracts, which are the main source of use by the population. This lack of data allows new research of this nature to be developed to contribute to the chemical and biological knowledge of these species.

*Lippia sidoides*, known as rosemary in northeastern Brazil, is among the aromatic species used in Brazilian Federal Government programs, such as RENISUS (National List of Plants of Interest to the Unified Health System—SUS), due to its medicinal properties [4,5]. Ethnobotanical data demonstrate that species of the *Lippia* genus have great versatility in local communities, particularly for treating Central Nervous System (CNS) disorders such as stress, anxiety and insomnia, and for use as tranquilizers [6].

Epilepsy is a CNS disorder that affects thousands of people of all ages worldwide. Epileptic seizures are characterized by recurrent convulsions, with cognitive, neurobiological and psychosocial damage as the main consequences [7]. The International League Against Epilepsy has introduced new definitions for the classification of the different forms of the disease and their mechanisms, based on recent advances in the field of medicine. This classification is grounded in signs and symptoms observed in electroencephalography, as well as in the behavior of brain activity [8].

Considering the different forms of epilepsy presentation, approximately 70% of epileptic patients respond well to treatment with anticonvulsants. However, a significant number of individuals experience uncontrolled seizures that cannot be managed with available medications alone. Although current medications prevent and reduce seizures, many adverse effects limit their use [9]. Despite efforts to develop medications to control frequent seizures, the negative response for some patients has resulted in serious medical, social and economic consequences, such as physical injuries and even death [10].

Alzheimer’s disease (AD) represents another global public health problem. This disease is characterized by a deficit in cholinergic transmission and the formation of senile plaques containing beta-amyloid protein in the brain. Among the treatments used for AD symptoms, acetylcholinesterase inhibitors are commonly employed [11]. Medications such as tacrine, donepezil, rivastigmine and galantamine are widely used; however, they only act on the symptoms and do not modify the course of the disease. These medications have different side effects, ranging from muscle cramps to gastrointestinal problems, which reflects the problem with their common use among patients [12].

Although medications that treat epilepsy and AD are well accepted by the medical community, many undesirable effects are observed in patients. Decreased cognitive abilities and psychiatric effects are the most relevant in patients who use medications to treat seizures [10]. Associated with drug treatment, complementary and alternative medicine presents treatments with natural products to control diseases associated with the CNS [13], and this option is promising in the context of alternative and effective treatments.

Based on the problems associated with using medications for epilepsy and AD, the scientific community is seeking new alternatives through medicinal plants to find different, safer and more effective therapeutic approaches that address symptoms and can help alleviate the disease. Associated with this and the therapeutic potential that the species *L. sidoides* presents, this study offers, for the first time, data that demonstrate the capacity of the extract of *L. sidoides* to act in the delay of convulsive crises and to act in the inhibition of the enzyme acetylcholinesterase. The data obtained indicate that, in the future, alternative therapies with *L. sidoides* extract may be considered as a complementary form of treatment for diseases that affect the CNS.

## 2. Methods

### 2.1. Plant Material and Obtaining Extracts

The leaves of *Lippia sidoides* were collected in the medicinal plant garden of the Regional University of Cariri-URCA, with latitudinal coordinates of 7°14′20.1″ S and longitudinal coordinates of 39°24′53.1″ W. The collection was carried out in March 2019 at 9 a.m. A sample containing the reproductive part (flowers) of the species was deposited in the Herbarium Caririense Dárdano de Andrade Lima at university, under registration number 3038.

To obtain the extract, the fresh leaves were cut and macerated with hexane to remove lipid components and then subjected to agitation with water in a refrigerated Shaker incubator (NT 715 novatecnica, São Paulo, Brazil) adopting the following parameters—temperature: 50 °C; rotation: 180 rpm; agitation time: 4 h/day—for 7 days. The yield corresponded to 4 L of aqueous solution, which was dried by atomization in Mini-spray dryer MSDi 1.0 equipment (Labmaq do Brasil), using a 1.2 mm spray nozzle, under the following operating conditions: (a) flow control: 200 mL/h; (b) inlet temperature: 120 ± 2 °C; (c) outlet temperature: 88 ± 2 °C; (d) atomization air flow: 45 L/min; and (e) blower flow: 1.80 m^3^/min.

### 2.2. Characterization of the Standardized Extract Produced by Spray Dryer

The quality of the powder sample was evaluated in relation to the following parameters: gross and compaction density, Hausner factor (indicator of powder flowability) and Carr index (measures the compressibility and flowability of a powder).

Gross density was determined by measuring the mass of the extract occupied in a 10 mL graduated cylinder. Compaction density was measured using 0.5 g of the sample in a 10 mL graduated cylinder that was subsequently subjected to maximum vortex agitation for 1 min. The result was calculated by the ratio between the volume occupied by the sample and the initial mass added to the graduated cylinder, according to the following equation (Equation (1)) [14,15].

*db* = *Ma*/*Vb* *dc* = *Ma*/*Vc* (1)

where *db* and *dc* are the gross and compaction density, respectively; *Ma* is the mass of dry extract; and *Vb* and *Vc* are the volume occupied by the mass of dry extract before and after compaction, respectively.

The Carr index (CI) and Hausner factor (HF) were calculated according to the equations (Equation (2)) below:

IC = *db* − *dc* × 100/*dc* FH = *dc*/*db* (2)

### 2.3. Chemical Characterization

The chemical characterization of the aqueous extract was previously published by this same research group, considering the same analysis conditions as this current work. It was performed using UPLC-ESI-MS/MS-system Shimadzu (Shimadzu Scientific Instruments Inc., Columbia, MD, USA) in negative ionization mode, where the predominance of phenolic compounds, especially flavonoids, was verified, confirming the occurrence of the same class of metabolites in the quantification of total flavonoids [16].

### 2.4. Biological Tests

#### 2.4.1. Zebrafish

Wild-type adult zebrafish (ZFa) aged 60 to 90 days (0.4 ± 0.1 g) of both sexes were obtained from a commercial supplier (Fortaleza, CE, Brazil). The animals were kept in a glass aquarium (n = 5/L) at a temperature of 25 ± 2 °C, in a 24 h light–dark cycle. Water was treated with antichlorine. The bioassays performed are in accordance with the Ethical Principles of Animal Experimentation and were approved by the Ethics Committee for the Use of Animals (CEUA) of the State University of Ceará (04983945/2021). After the experiments, the animals were sacrificed by freezing by immersing in ice water (2–4 °C) for 10 min until loss of opercular movements [17].

#### 2.4.2. Pentylenetetrazol (PTZ)-Induced Seizure

The animals (n = 6/group) were treated with *L. sidoides* extract that had anxiolytic effect (400 mg/kg; 20 μL; i.p.), DZP (10 mg/kg; 20 μL; i.p.) and vehicle (DMSO 3%; 20 μL; i.p.). After 1 h, the animals were individually exposed to 7.5 mM PTZ, dissolved in water in a 250 mL beaker, and the three-stage seizure behavior was evaluated: stage I, drastic increase in swimming activity; stage II, whirlpool behavior; and stage III, clonus-like convulsions followed by loss of posture when the animal fell to the side and remained motionless for 1–3 s. At the end of the evaluation of the three stages of the test, the animals were euthanized on ice.

#### 2.4.3. Acetylcholinesterase Inhibitory Activity

The acetylcholinesterase inhibition assay was determined by the method described in Ellman et al. (1961) [18]. The following solutions were added to 96-well plates: 25 µL Acetylcholine iodide (15 mM), 125 µL 5,5-dithiobis-[2-nitrobenzoic acid] in Tris/HCl solution (50 nM, pH = 8, with 0.1 M NaCl and 0.02 M MgC_l2_ 6H_2_O (Ellman’s reagent), 50 µL of Tris/HCl solution containing bovine serum albumin, and 25 µL of the extract at a final concentration of 2 mg/mL.

Absorbance was measured for 30 s at 405 nm, and then, 25 µL of the enzyme acetylcholinesterase was added, and the absorbance was read every minute for 30 min. Absorbance values for the extract were determined, and the percentage of acetylcholinesterase inhibition was calculated by comparing the reaction rates (substrate hydrolysis) of the samples in relation to the blank. The standard used as a control was physostigmine, and the tests were performed in triplicate.

#### 2.4.4. Osmotic Fragility in Erythrocytes

##### Preparation of Human Erythrocytes

The erythrocyte samples were donated from the blood bank of the Laboratory of Clinical Analysis of Biomedicine. The method developed was reviewed and approved by the committee of clinical analysis of biomedicine of the Doctor Leão Sampaio University Center. The procedures were performed according to the manual of laboratory immunohematology-Ministry of Health, Brazil, and by the ARRIVE guidelines.

The blood used was type O+, which was initially homogenized in sodium citrate before the procedure. In a test tube, 900 µL of saline solution was pipetted, followed by 100 µL of whole blood in sodium citrate. The red blood cell washing procedure was performed by centrifuging the tube at 3500 rpm for 15 s, discarding the supernatant at the end of each centrifugation. The washing process was repeated 6 times, removing as much of the supernatant as possible with absorbent paper in the last wash. Finally, the red blood cells were homogenized with 900 µL of saline solution [19].

##### Osmotic Fragility

For osmotic fragility, the lowest concentration of the extract with hemolytic capacity (1000 µg/mL) was chosen, testing different concentrations of NaCl (0.12%, 0.24%, 0.36%, 0.48%, 0.60%, 0.72% and 0.90%). The negative control consisted of each tested concentration of NaCl without the extract. The percentage of hemolysis was calculated, and the “fragility curves” were plotted using the percentage of hemolysis (% hemolysis) for each NaCl concentration (relative to the 100% hemolysis tube with 0.12% NaCl), according to Equation [19]:

% hemolysis = Abs_sample_ − Abs_blank_ × 100/Abs_control_

where ABs_sample_ represents the absorbance of the sample, Abs_blank_ represents the absorbance of the blank test, and Abs_control_ represents the absorbance of the control.

#### 2.4.5. Statistical Analysis

All analyses were performed in triplicate, and data were expressed as mean (*n* = 3) ± Standard Deviation (S.D.) using one- and two-way Analysis of Variance (ANOVA) followed by Tukey’s test for multiple comparison for data with normal distribution and significantly similar standard deviations with values of *p* < 0.05; *p* < 0.01 and *p* < 0.001. All analyses were performed with GraphPad Prism software v. 8.01.

## 3. Results and Discussion

### 3.1. Characterization of the Standardized Extract Produced by Spray Dryer

The total mass of the extract after drying was 77.60 g, offering a yield of 43.11%. The compaction and fluidity values are shown in Table 1.

### 3.2. Anticonvulsant Effect

The experimental model used in this study provides safety for the data presented, due to the fact that the Zebrafish genome is 70% homologous to that of humans and has a well-known CNS anatomy. Thus, the experimental model is satisfactory for behavioral, toxicological and general disease analyses that affect the CNS [20].

**Figure 1:**
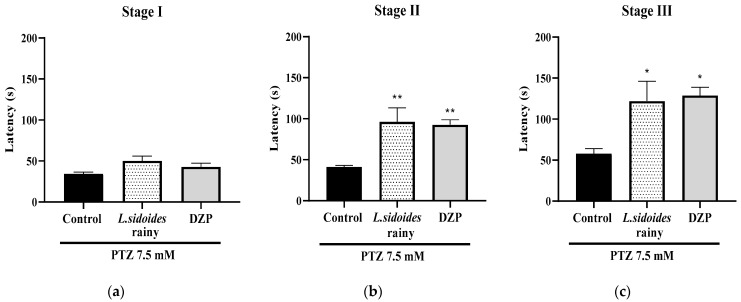
Effect of *L. sidoides* extracts on PTZ-induced seizure in adult zebrafish. (**a**) Stage I extract, (**b**) Stage II extract, (**c**) Stage III extract. DZP—Diazepam (10 mg/kg; 20 µL; i.p.). Control: Vehicle—DMSO 3% (20 µL; i.p.). Values represent the mean ± standard error of the mean (S.E.M.) for 6 animals/group. ANOVA followed by Tukey (* *p* < 0.05, ** *p* < 0.01 vs. DZP).

The anticonvulsant effect of L. sidoides extract demonstrated that the concentration of 400 mg/kg delayed the latency of convulsive seizures in the three stages (** *p* < 0.01; * *p* < 0.05; *p* < 0.05 vs. Control), similar to DZP (Figure 1). In the three stages, an increase in the latency time for the onset of PTZ-induced convulsive crises was observed, suggesting a convulsant action of the extract with an effect similar to that of the drug diazepam.

A similar effect was observed for the ethanolic extract of L. citriodora, which showed dose-dependent anticonvulsant action starting at 200 mg/kg [9]. Based on the chemical analysis of both studies, the presence of compounds belonging to the flavonoid class can be noted.

The action of drugs used to prevent seizures occurs through interaction with GABAa receptors. This mechanism of action has been demonstrated in other studies that treated animals with natural products and derivatives [21]. Several flavonoids have good affinity for interaction with GABAa receptors. Among these are luteolin and apeginin, which, in low concentrations (28.66 μM and 2.52 μM, respectively), can bind to the receptor, which may be one of the mechanisms of action by which extracts containing these compounds act in delaying convulsive crises [22].

Albizia adianthifolia extract containing apegenin in its composition demonstrated protection in mice against convulsions [23]. This flavonoid was also found in *L. sidoides* extract [16], and its anticonvulsant effect may be associated with this compound. In addition to apegenin, the flavonoids luteolin and narigenin may be associated with this activity.

According to [24], phenolic compounds such as quercetin are capable of reducing the prolonged state of convulsion at concentrations starting from 10 mg/kg in the PTZ model. In this sense, the effect observed for the *L. sidoides* extract may be associated with the presence of the different flavonoids that were identified in its chemical composition [16].

The delay in seizure latency was demonstrated for the dry ethanolic extract of *L. alba* and for the essential oil of *L. origanoides*. The *L. alba* extract presented in its composition 40% of flavonoids with which the delay in seizures and the relaxant effect of the extract were associated. Animals treated with *L. origanoides* essential oil did not develop generalized seizures; these findings were associated with the presence thymol in the chemical composition [25,26].

These results confirm the potential effect *Lippia* species on CNS diseases, with flavonoids possibly being the main agent responsible for this activity.

### 3.3. Anticholinesterase Effect

This study is the first report on the inhibitory effect of the acetylcholinesterase enzyme by the aqueous extract of *L sidoides*.

**Figure 2:**
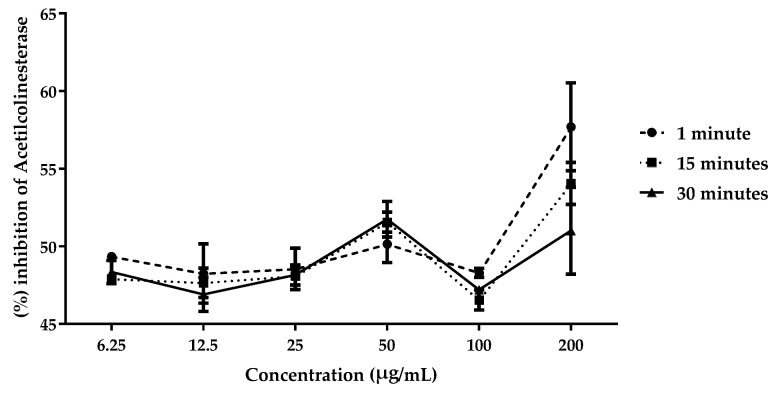
Inhibition of the enzyme acetylcholinesterase by the aqueous extract of *L. sidoides*.

Acetylcholinesterase inhibitors increase cholinergic levels in the brain by preventing the degradation of acetylcholine by the enzyme acetylcholinesterase. The extract showed low inhibitory activity against the enzyme at the initial concentrations tested; however, an increase in enzymatic inhibition was observed at a concentration of 200 µg/mL. It was observed that, over time, the activity tended to decrease, suggesting a reversible action by the extract (Figure 2). The IC_50_ calculation confirmed the oscillation in activity over time (Table 2).

The reversible effect promoted by *L. sidoides* extract on enzyme inhibitory activity is common to drugs used to treat AD. In contrast, it has been demonstrated that *L. sidoides* extract has a safety profile at high doses tested when evaluated by different toxicity methods [16]. This is an advantage to be considered in new studies with the species.

Different mixtures of *L. alba* essential oil showed inhibitory effects on acetylcholinesterase with inhibition zones similar to those of physostigmine. The different chemotypes were evaluated at concentrations of 100 μg/mL, 50 μg/mL, 25 μg/mL, 12.5 μg/mL, 6.25 μg/mL and 3.12 μg/mL [27]. Another study showed the inhibitory effect of acetone extract with *L. janvica* leaves on acetylcholinesterase, where the concentration of 500 μg/mL is capable of inhibiting more than 50% of the enzymatic activity; for this extract, a value of 80.72 ± 4.93 mg QE/g in the total flavonoid content was reported, which may be associated with anticholinesterase activity [28].

Acetylcholinesterase inhibition was verified in different fractions obtained from the species *L. salsa*. Among the results, better inhibition profiles were observed for the fraction rich in apigenin and luteolin, two flavones of clinical interest [29]. These findings corroborate the data of this study, since the presence of these two constituents was also demonstrated for *L. sidoides* [16].

Infusions of different species have a significant effect on the inhibition of the enzyme acetylcholinesterase. The results are associated with the content of phenolic acids and flavonoids found in the species [30]. Among the compounds found, the presence of luteolin was revealed, which was also identified in this work. These findings corroborate the idea that the flavonoids present in the *L. sidoides* extract are responsible for the anticholinesterase effect of the extract.

### 3.4. Protective Effect of Extract on Osmotic Fragility in Erythrocytes

Among the different forms of AD progression, oxidative stress is one of the main contributors to the progression of the disease. Thus, therapies that present combined actions aimed at improving symptoms and reducing disease progression are necessary to improve the patient’s quality of life [31].

**Figure 3:**
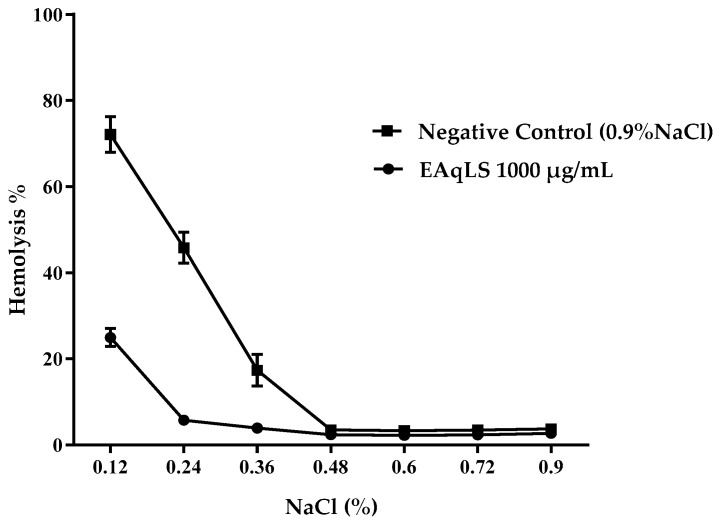
Osmotic fragility of blood samples treated with 1000 μg/mL of the aqueous extract of *Lippia sidoides* at different NaCl concentrations.

In the osmotic fragility test, the *L. sidoides* extract protected the cells from hemolysis in all variations of NaCl concentrations (Figure 3). Analyzing Figure 3, it is possible to observe that at a concentration of 0.12% NaCl (hypotonic), the extract was able to protect the cell, with a hemolysis rate of less than 50%. At the other NaCl concentrations, the extract had a similar effect to the negative control, indicating a hemolysis rate close to zero.

Aqueous extracts of various species, including *Lippia* species, were tested for the protection of erythrocyte membranes subjected to hypotonic shocks with NaCl. The extracts reduced membrane lysis in relation to the control group, decreasing the values of the cell mortality rate and intensity [32].

Several flavonoids are involved in the protection of blood cells, especially through their antioxidant effects [33,34]. Studies show that preliminary treatment of erythrocytes with flavonoids prevents deformation of the erythrocyte membrane [35].

This result may be related to the presence of compounds from the apegenin, luteolin and naringenin that are found in the extract of the species under study [16]. The authors in [36] relate the antihemolytic activity to the presence of phenolic compounds that may relate to the cell membrane and confer protective effects.

Flavonoids have antioxidant properties with varied mechanisms of action. These compounds can act by inhibiting reactive species or preventing their formation through electron donation [37]. The protective effect of the aqueous extract of *L. sidoides* may be related to its antioxidant capacity conferred by the presence of phenolic compounds.

In the study by [38], the aqueous extract of *L. sidoides* was tested for its total antioxidant capacity with a significant response at concentrations of up to 1 mg/mL. In another study, the antioxidant effect of *L. sidoides* extract was evaluated by the DPPH test, demonstrating a significant result with 50% radical inhibition at a concentration of 5 µg/mL. The antioxidant action was attributed to the presence of flavonoids and coumarins [39].

The evaluation of the protective property of extracts in biological membranes should be analyzed as a way of demonstrating possible toxic effects that these extracts may have when used for tests with biological activity. From these results, it was evident that the *L. sidoides* extract acts in the progressive protection of the erythrocyte membrane when exposed to different saline concentrations.

## 4. Conclusions

The aqueous extract of *Lippia sidoides* has an anticonvulsant effect, delaying convulsive crises. In the anticholinesterase assay, dose-dependent action and variation in effect over time were observed, demonstrating the reversible nature of the extract’s effect. In the osmotic fragility test, the extract showed satisfactory results, as it provided cellular protection across all variations of NaCl concentration. These results are promising for the development of new drugs based on natural products that have a broad spectrum of action, are effective and have a low incidence offside effect.

From this perspective, this study has proved to be the basis for the development of future tests that can investigate the mechanisms of action involved in the anticonvulsant effects of *L. sidoides* extract and thus be able to proceed to the development of a product with such a property.

## Figures and Tables

**Table 1:** Physicochemical quality values of the aqueous extract of *L. sidoides*.

Gross density	0.20 g/mL
Compression	0.24 g/mL
Carr Index	20%
Hausner factor	0.83

**Table 2:** IC_50_ values of acetylcholinesterase inhibition by the aqueous extract of *L. sidoides*.

IC_50_	1 min	15 min	30 min
	28.58 ± 0.219	61.28 ± 0.273	160.90 ± 0.649

## Data Availability

The data set used to prepare this article will be made available upon consultation with the authors.
